# Supplementary material for: Evolution of Shh endoderm enhancers during morphological transition from ventral lungs to dorsal gas bladder
Source: Nat Commun. 2017 Feb 3;8:14300. doi: 10.1038/ncomms14300 (PMC5296767; doi:10.1038/ncomms14300)
Supplement: Supplementary Information — Supplementary Figures 1-11 and Supplementary Tables 1-4 [file ncomms14300-s1.pdf]

Supplementary Figure 1

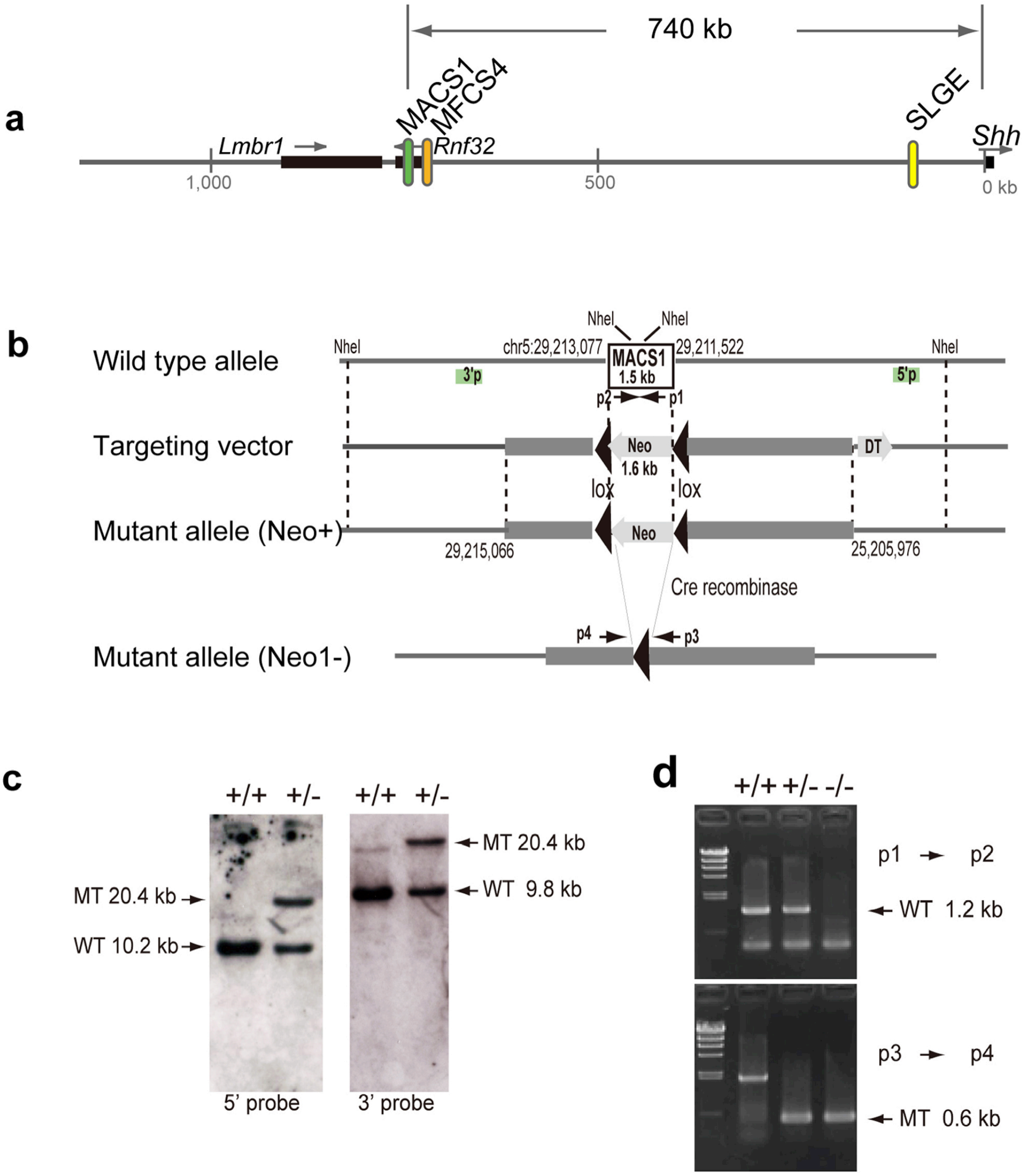

Supplementary Fig. 1. Generation of the MACS1 knockout mouse. Genomic location of three endoderm enhancers in the *Shh* regulatory region (a). Coloured bars mark MACS1 (green), MFCS4 (orange) and SLGE (yellow). MACS1 is localized at the 740 kb upstream of the *Shh* TSS (0 kb) in the mouse genome. Schematic diagram of ES cell targeting of MACS1 (b). Southern blot

analysis of the mutant ES cell clone (c). Southern blot analysis was carried out for NheI-digested genomic DNAs. A 5' probe (5'p in (b)) detected bands of 10.2 kb for the wild type allele (WT) and 20.4 kb for the mutant allele (MT). A 3' probe (3'p in (b)) detected bands of 9.8 kb for WT and 20.4 kb for MT. PCR primer pairs p1-p2 and p3-p4 in (b) (Supplementary Table 3) were used to detect 1.2 kb WT and 660 bp MT amplification products, respectively (d).

Supplementary Figure 2

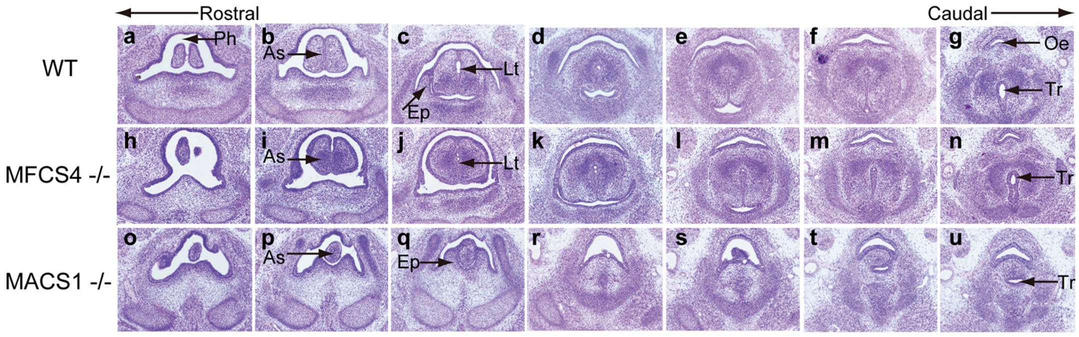

Supplementary Fig. 2. Defects of the respiratory organs in MFCS4-/- and MACS1-/- embryos. Serial transverse sections of wild type (a-g), MFCS4-/- (h-n) and MACS1-/- (o-u) embryos at E13.5. In MFCS4-/- embryos, the epiglottis is truncated (i, j), but the arytenoid swelling is bifurcated (h, i) and the laryngotracheal groove is well formed (j-l). In MACS1-/- embryos, the epiglottis is recognizable (p, q), but the arytenoid swelling is hypoplastic and not bifurcated (o-q). Consequently, the laryngotracheal groove is not formed (q-s) in the MACS1-/- embryos. The septum between oeophagus and larynx is deficient (t) but the trachea is formed (u). As, arytenoid swelling; Ep, epiglottis; Oe, Oesophagus; Lt, laryngotracheal groove; Ph, pharynx; Tr, trachea.

Supplementary Figure 3

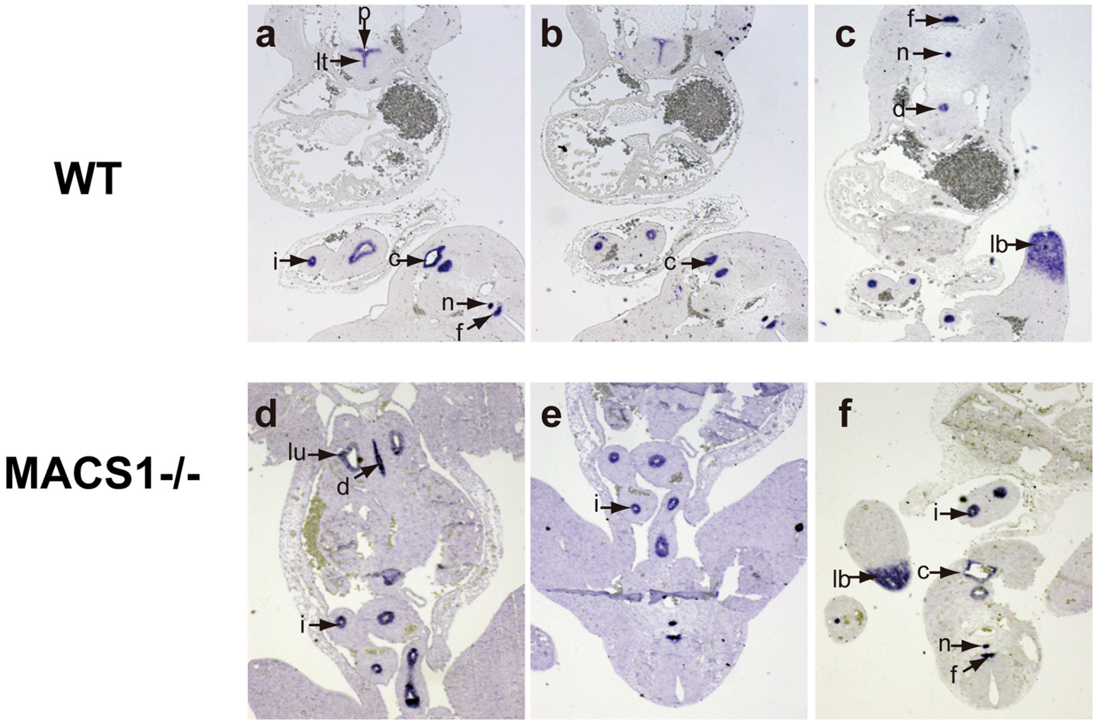

Supplementary Fig. 3. *Shh* expression in the lung and gut of MACS1-/- embryos. Serial sections of wild type (a-c) and MACS1-/- (d-f) embryos at E11.5. *Shh* expression is normally detected in the epithelia of lung, gut and urogenital tract of MACS1-/- embryos. c, cloaca; d, digestive tube; f, floor plate; i, intestine; lb, limb bud; lt, laryngotracheal groove; lu, lung bud; n, notochord; p, pharynx.

Supplementary Figure 4

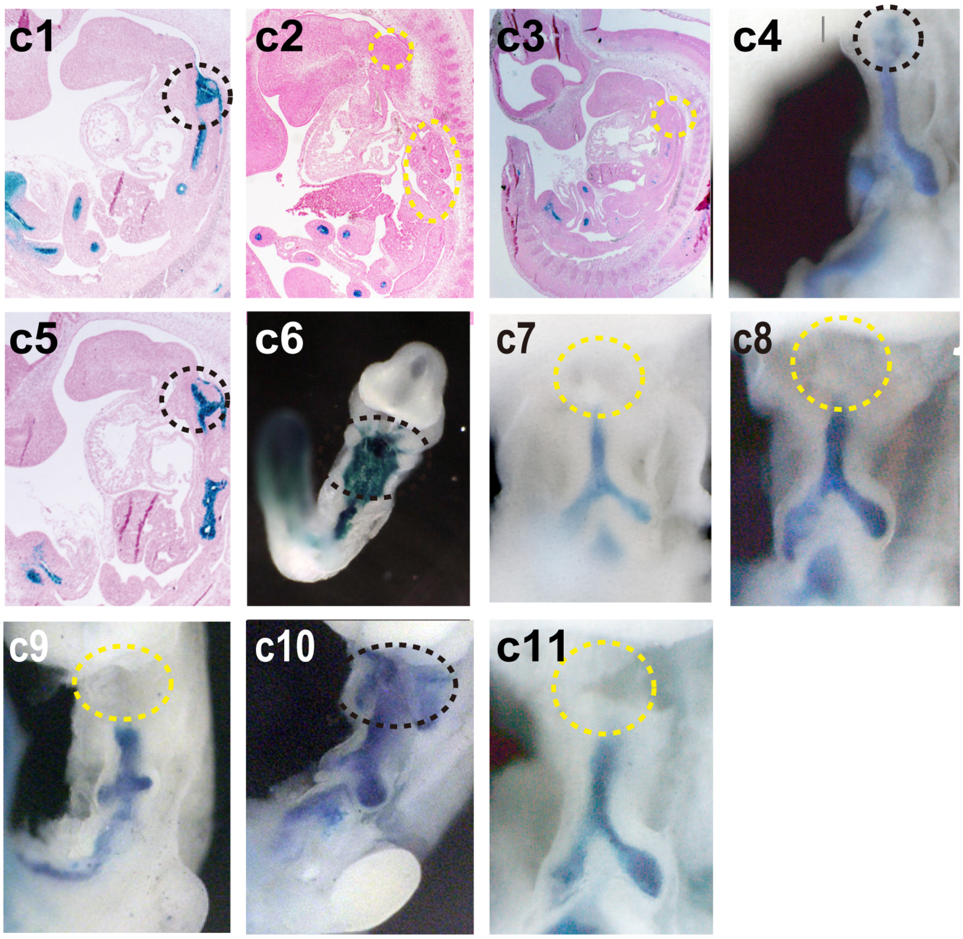

Supplementary Fig. 4. *LacZ* reporter expression driven by serial deletion constructs. The label of each image matches that of the corresponding construct in Fig. 3. Fully active constructs (Fig. 3a) can drive *LacZ* reporter expression in the epithelia of larynx, lung and gut from hind stomach to urogenital tract (c1, c4 to c5, c-10). Dashed black lines enclose *LacZ* reporter expression in the laryngeal epithelium, while dashed yellow lines denote abrogation of *LacZ* expression in the laryngeal epithelium.

Supplementary Figure 5

```
                    HNF4-Q6                                      CMAF
                        HNF4-DR1                       CDP
Mouse           TAGGTGAACTCTGTCTGCTC-A-GG----AGAGTCCT--AAGTGATCCATCTA-ATGT
Rat             TAGGTGAATTCTGTCTGCTC-A-GG----AAAGTCTT--AAGTGATCCATCTC-ATGT
Cow             CAGGTGAACTCTGCCTGCCC-A-GG-----GGCCCCTCTGAGC-ATCCATCTTGATGT
Sheep           CAGGTGAACTCTGCCTGCCC-A-GG-----GGCCCCTCTGAGC-ATCCATCTTGATGT
Horse           CAGGTGAACTCTGTCTGCCC-A-GG----AGGCCTCTCCAAGTGATCCATCTTGATGT
Human           CAGGTGAACTCTGTCTACTC-A-GG----AGGGCTTTTTAAGTGATCCATCTTGATGT
Opossum         CAGGTGAACTTTGTCTAGCA-A-GG----AGACCTTTTAAAGTGATCCATCTTGATGT
Platypus        CAGGTGAACTTTGTCCACCA-GTGG----AGGCCTTCCTAAGTGATCTATCTTTGTGT
Chicken         GGCGTGAACTTTGTCCAC---AGGG----AAGCCTTTTAAAGTGATCCA-----GTGT
Turkey          GGCATGAACTTTGTCCAC---AGGG----AAGCCTTTTAAAGTGATCCA-----GTGT
Alligator       TTGGTGAACTTTGNCCAT---AGGG----AAGTCTT-TTGAGTGATCCATCTTAATGT
Turtle          TTAGTGAACTCTGT--AC---AGAG----AAGCCTTCTTAAGTGATCCATCTTAATGT
Lizard          GAGATGAACTTTGCCTGCCA-AAGGCA--AGCCCTTTTAAAGCAATCTATCTTTATGT
Xenopus         --GGTGAACTTTGTGCACTT-TGGGGAGTAGTTCTTTGCAAGTAATCCATCTTAATGT
Coelacanth      CAGGTGAACTTTGTCCACC--ACAG---GGTGACTTTTCAAGTGATCCATCTAAATGT
Spotted gar     CAGGTGCACTTTGTCTATCTTACAG---GAGGCCTTTCAAAGCAATCCATCTTGGTGT
Skate           TTGGTGGACATTGCTCATT----------------TTAGAAAACTTTCATTTAAATGC
Elephant shark  TTAGTGGACTTTGCCCACC----------------CCTGAAAACTTTCATTTAAATGC
                    ** *   **                           *    *  *      **

                                                                     HSF
                    CMAF                 AP1                        OCT1
                    HMGIY          Fox                   BARBIE
Mouse           C-AGCAAATTGCTTACTAATGTGTTGACTTAGGGCCAC-CCTCAAAAGCAAAATGAAA
Rat             C-AGCAAATTGCTTACTAATGTGTTGACTTAGGGCCAC-TCTCAAAAGCAAAAAGAAA
Cow             C-AGCAA-TTGCTTACTAATGTGTTGACTTACGGCTGC-CCTCAAAAGCAAAGCGAAA
Sheep           C-AGCAA-TTGCTTACTAATGTGTTGACTTACGGCCGC-CCTCAAAAGCAAAGTGAAA
Horse           C-AGCAAATTGCTTACTAATGTGTTGACTTATGGCCAC-CCTCAAAAGCAAAATGAAA
Human           C-AGCAAATTGCTTACTAATGTGTTGACTTATGGCCGC-CCTCAAAAGCAAAATGAAA
Opossum         C-AGCAAATTGCTTACTAATGTGTTGACTTATGGAAAC-TCTCTAAAGCAAAATGAAA
Platypus        C-AGCAAATTGCTTACTAATGTGTTGACTTATGGGAAC-CCTCCAAAGCAAAATGAAA
Chicken         C-AGCAAATTGCTTACTAATGTGTTGACTTATGGCAAC-CCTCAAAAGTAAAATGAAA
Turkey          C-AGCAAATTGCTTACTAATGTGTTGACTTATGGCAAC-CCTCAAAAGTAAAATGAAA
Alligator       C-AGCAAATTGCTTACTAATGTGTTGACTTACGGCAAC-CCTCAAAAGTAAAATGAAA
Turtle          C-AGTAAATTGTTTACTAATGTGTTGACTTATGGCAAC-CCTCAAAAGGAAAATGAAA
Lizard          C-AGCAAATTGCTTACTAATGTGTTGACTTA--------------AAAGTAAAATGAAA
Xenopus         C-AGCAAATTGCTTACTAATGTGTTGACTTATGGCAAT-CCTCAAAAGCAAAATGGAA
Coelacanth      C-AGCAAATTGCTTACTAATGTGTTGACTTATGGCAAG-CCTCAAAAGTAAAATGAAA
Spotted gar     C-AGCAAATTGCTTACTAATGTGTTGACTTATGGTAGGACCTTAAAAGTAAAATGAAA
Skate           CCAACAAATTGGTTACTAATGTGTTGACCTCCAGTACG-CCTCACAACTAACCTTAAA
Elephant shark  C-AGCAAATTGGTTACTAATGTGTTGACTTATGGTAA-GCCTCAAAACTAAACTGAAA
                * *  ** *** **************** *               **  **     **
                               29 bp

                     HSF
                OCT1
                           PAX5
Mouse           A--GCC-TGGAATTTATAGCATCTCAATGCATCAGCTG
Rat             A--GCC-TGGAATTTATAGCATCTCAATGCATCAGCTG
Cow             C--GCC-TGGAATTTACAGCGTATCAGTGAATCCGTTG
Sheep           C--GCC-TGGAATTTACAGCGTATCAATGAATCCGTTG
Horse           A--GCT-TGGAATTTACAGCATATCAATGAATCAGTTG
Human           A--GCC-TGGAATTTACAGCATATCAATGAATCAGTTG
Opossum         A--GGC-TGGAATTTACAGCATATCAATGAATCAGTTG
Platypus        A--GGT-TGGAATTTACAGCATATCAATGAATCAGTTG
Chicken         A--GGC-TGGAATTTACAGCATATCAATGAATCAGTTG
Turkey          A--GGC-TGGAATTTACAGCATATCAATGAATCAGTTG
Alligator       A--GGC-TGGAATTTACAGCATATCAATGAATCAGTTG
Turtle          A--GGC-TAGAGTTTACAGCATATCAATGAATCAGTTG
Lizard          A--GGC-TGGAATTTACAGCGTATCAATGAATCAGTTG
Xenopus         G--GGCCTGGAATTTACAGCAGATCTATGAATCGGC-G
Coelacanth      A--GGACTGGAATTTACAGCATATCAATGAATCAGTTG
Spotted gar     A--GGCTTGGTATTTACTGCATATCAAGGAATCAGTTG
Skate           ATAAACAAGGAATTTACAACGTATCAATGAA------G
Elephant shark  ---AGCAGGGAATTTACAACATATCTATGTA------G
                         *  ****   *   **   * *      *
```

Supplementary Fig. 5. Sequence comparison and prediction of transcription factor binding motifs in the MACS1 sequence. ClustalW and rVISTA analyses of a 140-bp sequence in mouse MACS1 (chr5: 29,212,687-29,212,597) that includes the 29-bp sequence (boxed) that is critical for laryngeal expression. We retrieved the genomic sequences from the UCSC and Ensembl genome browsers, and aligned the syntenic region of the 18 vertebrates using the ClustalW system (http://clustalw.ddbj.nig.ac.jp/). The rVISTA program (http://rvista.dcode.org) predicted a Fox binding motif in the 29-bp critical region. Perfectly conserved nucleotides are indicated by asterisk below the aligned sequences.

Supplementary Figure 6

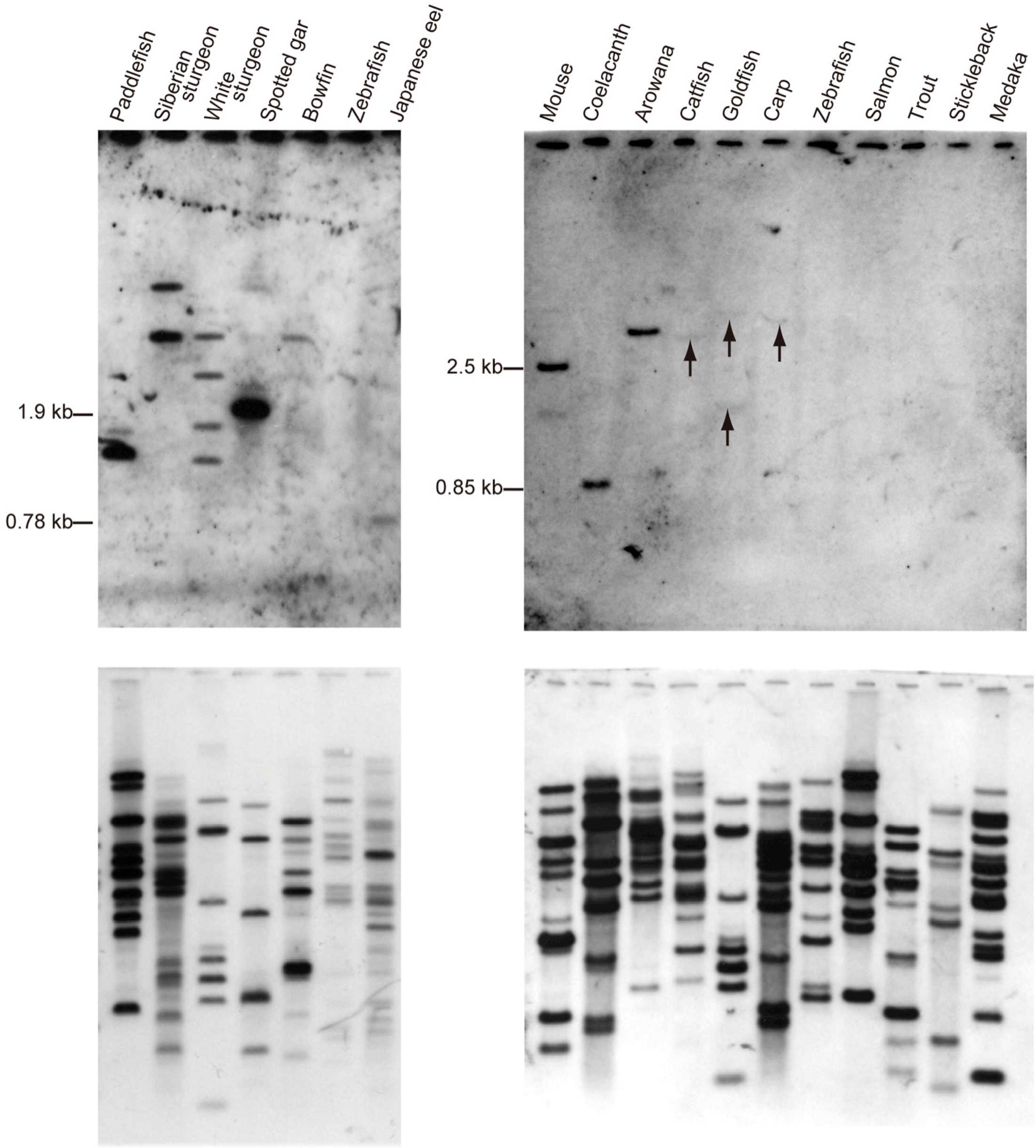

Supplementary Fig. 6. Southern blot analysis of MACS1 conservation. A spotted gar MACS1 probe was hybridized with the genomic DNAs (upper images). Genomic DNAs of mouse, a lobe-finned fish (coelacanth), five basal ray-finned fish (paddlefish, Siberian sturgeon, white sturgeon, spotted gar and bowfin) and two teleost fish (Japanese eel and alowana) yielded clear bands. Among teleost fish, catfish, goldfish and carp yielded very faint bands (black arrows). By contrast, zebrafish and euteleost fish (salmon, trout, stickleback and

medaka) did not yield any band. A mouse actin probe was used to examine the condition of the genomic DNAs (lower images).

Supplementary Figure 7

a

```
Medaka            GTTATCTCACAGCAATCCATCTCCATGTCGGTA-CCGTCTTCCCTAATGTGTTGACTCAG 59
Salmon            GTTGTCTCACCTCAATCCATCTTGATGTCAGCAGTTATCCTCCTTAATGTGTTGACTTAG 60
Spottd gar        GGCCTTTCAAAGCAATCCATCTTGGTGTCAGCAAAT-TGCTTACTAATGTGTTGACTTAT 59
                  *   * ***   **********   **** * *    *  *   ************* *

Medaka            GGTTGGACCTTCAAAAG 76
Salmon            GGTCGGACCTCAAAAAG 77                 51/77 (66%)
Spotted gar       GGTAGGACCTT-AAAAG 75
                  *** ******  *****
```

b

```
Spotted gar       GGCCTTTCAAAGCAATCCATCTTGGTGTCAGCAAAT-TGCTTACTAATGTGTTGACTTAT 59
Salmon            GTTGTCTCACCTCAATCCATCTTGATGTCAGCAGTTATCCTCCTTAATGTGTTGACTTAG 60
                  *   * ***   ************ ******** * * **   ***************

Spotted gar       GGTAGGACCTT-AAAAG 75
Salmon            GGTCGGACCTCAAAAAG 77                 58/77 (75%)
                  *** ******  *****
```

c

```
Medaka            GTTATCTCACAGCAATCCATCTCCATGTCGGTA-CCGTCTTCCCTAATGTGTTGACTCAG 59
Salmonn           GTTGTCTCACCTCAATCCATCTTGATGTCAGCAGTTATCCTCCTTAATGTGTTGACTTAG 60
                  *** ******  **********  ***** * *    ** *** ************* **

Medaka            GGTTGGACCTTCAAAAG 76
Salmon            GGTCGGACCTCAAAAAG 77                 60/77 (78%)
                  *** ******  *****
```

Supplementary Fig. 7. Sequence similarity of Block-1 between medaka and salmon or between salmon and spotted gar. The overall conservation of Block-1 among the three species is relatively low (a). Higher similarity is evident between spotted gar and salmon (b) and between medaka and salmon (c).

Supplementary Figure 8

a

```
Medaka_core1        GCTGTTGTGTTGAC--CACAATGTCCAGACTGTTTAAACTTAAACACAAATCTTTTGTCC 58
Salmon              GCTTTTGTGTTGACCCTGGAATGTCCAGACTGTTTGA-CCAAAACACACACTCTCCGTA- 58
Spotted gar         -TCTGTGTTTTGAC--TGGAATGTATAAGTTACTTGA-CTAGAACACACATTTCT--TA- 53
                         *** *****     *****  *   *  ** * *   ****** *       *

Medaka_core-1       ACAATAGTGGACAAACAGTGTATGATTCACAAAGCATCAAAGGTGCTCGACA 110
Salmon              --ATTAGTTTACAAACAGTGTATGATTCACATGGCATCAAAGGCCCTCTGAA 108  59/108 (55%)
Spotted gar         --ATTAGTTTACCAACAGTTTATGGTTCACACGGCATCAAAG-CCCTGTGGA 102
                      * ****  ** ****** **** ******  *********   **    *
```

b

```
Medaka_core-1       GCTGTTGTGTTGACCAC--AATGTCCAGACTGTTTAAACTTAAACACAAATCTTTTGTCC 58
Salmon              GCTTTTGTGTTGACCCTGGAATGTCCAGACTGTTTGA-CCAAAACACACACTCTCCGTA- 58
                    *** ***********    **************** * *  ******* *   *  **

Medaka_core-1       ACAATAGTGGACAAACAGTGTATGATTCACAAAGCATCAAAGGTGCTCGACA 110   79/108 (73%)
Salmon              --ATTAGTTTACAAACAGTGTATGATTCACATGGCATCAAAGGCCCTCTGAA 108
                      * ****  *********************  **********  ***   *
```

c

```
Salmon              GCTTTTGTGTTGACCCTGGAATGTCCAGACTGTTTGACCAAAACACACACTCTCCGTAAT 60
Spotted gar         -TCTGTGTTTTGAC--TGGAATGTATAAGTTACTTGACTAGAACACACATTTCT--TAAT 55
                       * *** *****  ********  *   *  ***** * ******** *     ****

Salmon              TAGTTTACAAACAGTGTATGATTCACATGGCATCAAAGGCCCTCTGAA 108    79/108 (73%)
Spotted gar         TAGTTTACCAACAGTTTATGGTTCACACGGCATCAAAG-CCCTGTGGA 102
                    ******** ****** **** ****** ********** **** ** *
```

Supplementary Fig. 8. Sequence similarity of core-1 in Block-2 between medaka and salmon or between salmon and spotted gar. The overall conservation of core-1 among medaka, salmon and spotted gar is relatively low (a). Higher similarity is evident between medaka and salmon (b) and between salmon and spotted gar (c).

Supplementary Figure 9

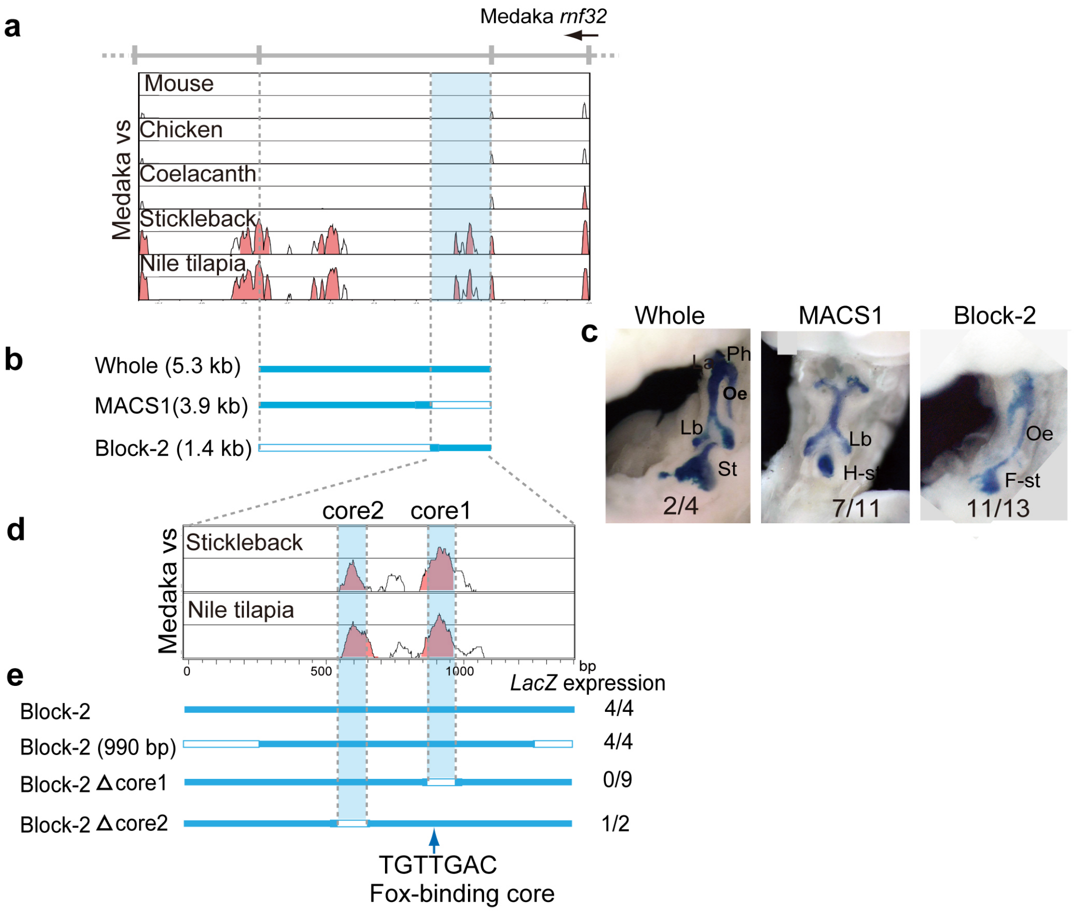

Supplementary Fig. 9. Transgenic reporter assay of the medaka enhancers in mouse embryos. VISTA plots of the partial sequence of *rnf32* in six vertebrate species (a). The medaka *rnf32* intronic sequence was used as the reference genome. A 5.3 kb medaka sequence (named Whole) has two conserved sequence blocks, Block1 (3.9 kb) and Block-2 (1.4 kb). The Whole fragment drove reporter expression in the epithelia of the oesophagus, forestomach and lung buds (c, left). Block-1 drove reporter expression in the epithelia of lung buds and hindstomach (c, middle). Block-2 drove expression in the epithelia of oesophagus and forestomach (c, right). Block-2 contains two euteleost-specific conserved core sequences, core1 and core2 (d, e). Transgenic reporter assay with deletion constructs showed that 990 bp in Block-2 is sufficient for enhancer activity (e). Deletion of core1 abrogated enhancer activity of Block-2 (e). On the other hand, loss of core2 did not affect

the enhancer activity of Block-2 (e). Oe, oeophagus; F-st, forestomach; H-st, hindstomach: La, larynx; Lb, lung bud; Ph, pharynx; St, stomach.

Supplementary Figure 10

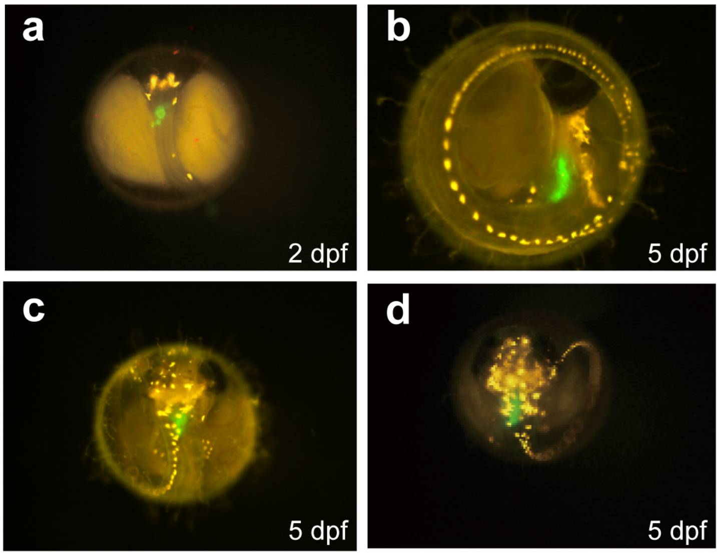

Supplementary Fig. 10. The Fox-binding motif in Block-2 is dispensable for reporter expression in medaka embryos. These images show the reporter expression driven by a Block-2 deletion construct that lacks nine bases (TGTGTTGAC) including the Fox-binding core (a red arrow in Supplementary Fig. 9e) in the four larvaes at 2 or 5 dpf. The construct drove similar reporter expression to that driven by intact Block-2 (Figure 6h).

Supplementary Figure 11

a

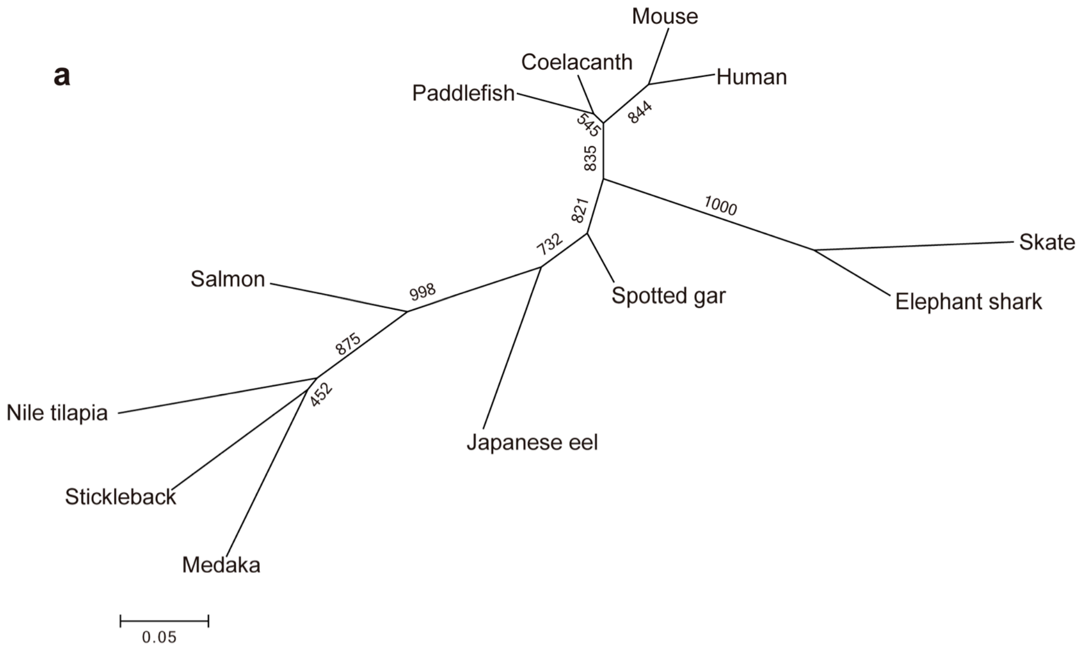

b

```
Skate            --GCTCAT-TTA-GAAAACTTTCATTTAAATGCCCAACAA--ATTGGTTACTAATGTGTT 54
Elephant shark   --GCCCAC-CCCTGAAAACTTTCATTTAAATGCC-AGCAA--ATTGGTTACTAATGTGTT 54
Coelacanth       GGGTGA-C-TTTTCAAGTGATCCATCTAAATGTC-AGCAA--ATTGCTTACTAATGTGTT 55
Paddlefish       GGGAGAGT-TTTTCAAGTGATCCATCTTAATGTC-AGCAA--ATTGCCTACTAATGTGTT 56
Human            -GGAGGGC-TTTTTAAGTGATCCATCTTGATGTC-AGCAA--ATTGCTTACTAATGTGTT 55
Mouse            -GGAGAG---TCCTAAGTGATCCATCT-AATGTC-AGCAA--ATTGCTTACTAATGTGTT 52
Spotted gar      -GGC---C-TTTCAAAGCAATCCATCTTGGTGTC-AGCAA--ATTGCTTACTAATGTGTT 52
Japanese eel     -GGGGAACGTTTCAAAGCAATCCGTCTTGGTGTC-AACAA--ATCACCTCCTAATGTGTT 56
Stickleback      --GTTA---TCTCACGGCTATCCATCTAGCTGTG-GGTAGT-GTCCCCCT-TAATGTGTT 52
Nile tilapia     --GTTA---TCTCGCAGCAATCCACGTAGTTGTC-GGAACT-GTCTCGAGGTAATGTGTT 53
Medaka           --GTTA---TCTCACAGCAATCCATCTCCATGTC-GGTACC-GTCTTCCC-TAATGTGTT 52
Salmon           --GTTG---TCTCACCTCAATCCATCTTGATGTC-AGCAGTTATCCTCCT-TAATGTGTT 53
                   *                 * *   *   **      *    *       *********

Skate            GACCTCCAGTACG-CCTCACAACTAACCTTAAA 86
Elephant shark   GACTTATGGTAAG-CCTCAAAACTAAACTGAAA 86
Coelacanth       GACTTATGGCAAG-CCTCAAAAGTAAAATGAAA 87
Paddlefish       GACTTATGGTAAGGCCTCAAGAGTAAAATGAAA 89
Human            GACTTATGGCCGC-C-TCAAAAGCAAAATGAAA 86
Mouse            GACTTAGGGCCAC-CCTCAAAAGCAAAATGAAA 84
Spotted gar      GACTTATGGTAGGACCTTAAAAGTAAAATGAAA 85
Japanese eel     GACTTATGGTAGGACCTGAAGAACAAAATAAAA 89
Stickleback      GACTCAGGGTCGGACCTCCGAAGTGAG-TAAAA 84
Nile tilapia     GACTCAGGGTCGGACCTCCGAAACAGGACGAAA 86
Medaka           GACTCAGGGTTGGACCTTCAAAAGGGGGCAAAA 85
Salmon           GACTTAGGGTCGGACCTCAAAAA---GCTAAAA 83
                 ***     *     * *    *        ***
```

Supplementary Fig. 11. Phylogenetic tree of Block-1 core sequences of diverse vertebrate taxa. The tree was made using MEGA7.0.18 (a) on the bassis of ~80bp core sequences of the Block-1 olthologues of 12 vertebrate species, which were aligned with ClutalW (DDBJ)(b).

Supplementary Table 1. Viability of the MACS1-/- mutants

Age	Wild type	MACS1+/-	MACS1-/-
E12.5	7*	19	7
E18.5	8	14	4
Newborn	19	24	7
2 days old	4	16	0
1 month old	15	14	0

*Numeral depicts the living animals at the stage.

Supplementary Table 2. Defects in the MFCS4-/- and MACS1-/- at E18.5

	MFCS4-/-	MACS1-/-
soft palate	truncation	normal
hyoid bone	hypoplasia	relatively normal
epiglottis	truncation	recognizable
arytenoids	minor truncation	dysplasia
vocal fold	recognizable	dysplasia
thyroid cartilage	normal	dysplasia
cricoid cartilage	normal	dysplasia
trachea	normal	relatively normal

Supplementary Table 3. Used primer pairs in this study

For ESC targeting of MACS1		
	F	R
Long arm of targeting vector	TCTGCATGCATGAGCTGTAG	TAGCTACATGTCCGCTAAGG
Short arm of targeting vector	GAGCACTCAGTCAATGTGTG	GCATTCCAGACATGGTGTTC
Southern blotting 5'probe	GACATCCTCGTGTTGTCTTC	CTTGAGAGTCACAAGGAAGG
Southern blotting 3'probe	TATCTCATGCTCCCAGAAGG	CCTTTGAGTCATCCATCCTC
For detection of WT allele (p1, p2)	AGAACAGGCACTCTAGATCC	ATAACCAGGAGTCCAGGTAC
For detection of MT allele (p3, p4)	CTGCTGTCAAAGATGGGTAG	ACTGCTCTTGCTCTCTAGAG
For transgenic assay		
	F	R
1264-bp including MACS1	AGAACAGGCACTCTAGATCC	ATAACCAGGAGTCCAGGTAC
c1	AACTTTGCTAGCATCTGTCC	ATAACCAGGAGTCCAGGTAC
c2	AGAACAGGCACTCTAGATCC	CATCTGACGGATTGTTAGCC
c3	AGAACAGGCACTCTAGATCC	GGCCACCCTCAAAAGCAAAA
c4	AGCCAGTGTACTGTTTTCAA	AGCCAGTGTACTGTTTTCAA
c5	TTGTACTGGTGAGTGTCAGG	TAGGTGAACTCTGTCTGCTC
c6 (inverse PCR)	TTGTACTGGTGAGTGTCAGG	ATAACCAGGAGTCCAGGTAC
c7 (inverse PCR)	AGGGCCACCCTCAAAAGCAA	GCTGACATTAGATGGATCAC
c8 (inverse PCR)	TTAGGGCCACCCTCAAAAGC	CATTAGTAAGCAATTTGCTG
c9 (inverse PCR)	TGTTGACTTAGGGCCACCCT	GCTGACATTAGATGGATCAC
c10 (inverse PCR)	TAATGTGTTGACTTAGGGCC	GCTGACATTAGATGGATCAC
c11 (inverse PCR)	GTGGCCCTAAGTCGGCGCATT AGTAAG	CTTACTAATGGGCCCGACTTA GGGCCAC
Medaka whole	GAGACGCAGGTTCTTTGAGG	GTCGGACAAAGCTGTCATTC
Medaka MACS1 (Block-1)	GGGACCAGTGTTGAGAATGT	GTCGGACAAAGCTGTCATTC
Medaka Block-2	GAGACGCAGGTTCTTTGAGG	GGTGGTGATCAGACATGTTG
Medaka Δ Block-2 core-1 (inverse PCR)	ATGAGAGCAGTCAGGGGTGT	ACCATGTGCATG TTTAGGTC
Medaka Δ Block-2 core-2 (inverse PCR)	CTGATGGTCAGACATTTGTC	ATAACCAGAAACTGCACTGG
Coelacanth MACS1	GGGACCAGTGTTGAGAATGT	CCAACCAGGTGAACTTTGTC

Skate MACS1	GCCAGAGCATACAATGAAGG	CAAGTTGGTGGACATTGCTC
Elephant shark MACS1	TGACCGTGCCTCATCTGAAA	TTTCAATCTCGCAAAGTTTT
Paddlefish MACS1	AGCATGTAATCTCCTGTACG	TTGCTTGTGGCCAGATAGTG
Spotted gar MACS1	TGCTTGCATCCGAAGACAAG	CAAATGGGTGCCAGTTAAGC
Spotted gar Block-2	GTCCAAGCAGTGAAAGAAGG	GGAGACATTGATGATGAGCC
Japanese eel MACS1	AAGCTGTCACTCAACTCCTG	CTGAGCTGAGCATGGTGATT
Japanese eel Block-2	CCAGACCAGAGTGATTCATG	AATCACCATGCTCAGCTCAG

Supplementary Table 4. Genomic sequences used for VISTA plots

	Location of the sequence for VISTA analysis	Database
Skate	LC009631: 1-8,490	http:/www.ddbj.nig.ac.jp/index-j.html
Elephant shark	chr KI636064: 496,375-498,997	UCSC:Callorhinchus_milii-6.1.3/calMil1
Human	chr7: 156,640,748-156,677,124	UCSC: GRCh38/hg38
Mouse	chr5: 29,197,926-29,225,291	UCSC;GRCm38/mm10
Coelacanth	JH127332: 287,480- 327,976	UCS: Broad/latCha1
Paddlefish	LC128331: 1-4,298	http:/www.ddbj.nig.ac.jp/index-j.html
Spotted gar	LG9: 41,365,815-41,372,726	Ensembl; http:/www.ensembl.org/index.html
Golden arowana	ASM16246v1 scaffold 53: 2,092,949-2,209,529	Genbank: NW_017371970 GPS_014192443
Japanese eel	AVPY00000000.1<br>KI307787：59,997−72,360	http://www.zfgenomics.com/sub/eel
Atlantic salmon	chr ssa29: 9,829,141-9,839,046	GenBank: XM_014180668.1, NC_027328
Medaka	chr 2: 17,804,591 -17,816,721	UCSC; NIG/UT MEDAKA1/oryLat2
Stickleback	chrXXI: 9,044,862 -9,052,154	UCSC; Broad/gasAcu1
Nile tilapia	chrLG9: 13,010,056 13,027,674	UCSC; Broad oreNil1.1/oreNil2
